# Analysis of *Ensifer aridi* Mutants Affecting Regulation of Methionine, Trehalose, and Inositol Metabolisms Suggests a Role in Stress Adaptation and Symbiosis Development

**DOI:** 10.3390/microorganisms10020298

**Published:** 2022-01-26

**Authors:** Meryem Belfquih, Abdelkarim Filali-Maltouf, Antoine Le Quéré

**Affiliations:** 1 Laboratory of Microbiology and Molecular Biology (LMBM), Department of Biology, Faculty of Sciences, University Mohammed V in Rabat, Rabat 10100, Morocco; belfquih.meryem@gmail.com; 2 Laboratoire des Symbioses Tropicales et Méditerranéennes (LSTM), University of Montpellier, IRD, CIRAD, INRAE, Institut Agro, 34398 Montpellier, France

**Keywords:** *Ensifer aridi*, nitrogen-fixing symbiosis, trehalose, S-adenosylmethionine cycling, inositol catabolism

## Abstract

Isolated from desert, the nitrogen-fixing bacterium *Ensifer aridi* LMR001 is capable of survival under particularly harsh environmental conditions. To obtain insights in molecular mechanisms involved in stress adaptation, a recent study using RNAseq revealed that the RpoE2-mediated general stress response was activated under mild saline stress but appeared non-essential for the bacterium to thrive under stress and develop the symbiosis. Functions associated with the stress response included the metabolisms of trehalose, methionine, and inositol. To explore the roles of these metabolisms in stress adaptation and symbiosis development, and the possible regulatory mechanisms involved, mutants were generated notably in regulators and their transcriptions were studied in various mutant backgrounds. We found that mutations in regulatory genes *nesR* and *sahR* of the methionine cycle generating S-adenosylmethionine negatively impacted symbiosis, tolerance to salt, and motility in the presence of NaCl. When both regulators were mutated, an increased tolerance to detergent, oxidative, and acid stresses was found, suggesting a modification of the cell wall components which may explain these phenotypes and support a major role of the fine-tuning methylation for symbiosis and stress adaptation of the bacterium. In contrast, we also found that mutations in the predicted trehalose transport and utilization regulator ThuR and the trehalose phosphate phosphatase OtsB-encoding genes improved symbiosis and growth in liquid medium containing 0.4 M of NaCl of LMR001Δ*otsB*, suggesting that trehalose metabolism control and possibly trehalose-6 phosphate cellular status may be biotechnologically engineered for improved symbiosis under stress. Finally, transcriptional fusions of *gfp* to promoters of selected genes and expression studies in the various mutant backgrounds suggest complex regulatory interplay between inositol, methionine, and trehalose metabolic pathways.

## 1. Introduction

Primarily found in deserts with an ability to develop the symbiosis of plants native and adapted to stress, *Ensifer aridi* represents an interesting species to study genes and functions that may explain its particular adaptation, broad host range, and phylo-geographic distribution [1,2,3,4,5]. Next-generation sequencing technologies (NGS) have become accessible to study any organism at the genome-wide level. Comparative genomic analyzes of 6 *Ensifer aridi* strains isolated in Asia, Africa, or America from diverse legumes showed that accessory genes were most probably located on large plasmids acquired through horizontal gene transfers that probably enabled the bacterium to develop the symbiosis with local hosts [6].

Among the most rapid methods to identify genes and functions associated to a particular stress, transcriptomics represents a method of choice. Genome-wide transcriptional responses resulting from abiotic stresses have been studied using microarray technology or NGS-based RNAseq in several rhizobial genera including, for example, *Bradyrhizobium* [7], *Rhizobium* [8,9], *Mesorhizobium* [10,11,12,13,14], or *Sinorhizobium* (*Ensifer*) [15,16,17,18,19,20]. The bacterial response to an osmotic upshift is complex and varies according to the strain and species. Nevertheless, to counteract osmotic pressure and the resulting oxidative stress, bacteria commonly accumulate osmoprotectants, compatible solutes, chaperones, and enzymes involved in ROS scavenging and they modify their cellular membranes and envelops [21].

According to a transcriptome analysis performed in *E. aridi* to explore the response of this bacterium to hyperosmotic stress, a number of genes were found differentially regulated, including the alternative extra-cytoplasmic function sigma factor RpoE2 which suggested activation of the general stress response (GSR) [15]. However, the mutation of the *rpoE2* gene suggested that the response was complex and that alternative regulatory mechanisms may be recruited by the bacterium to cope with various stresses. Among genes that were found differentially regulated, a strong repression of motility genes could be identified and validated phenotypically by swimming assays [15]. We also found that salt stress altered regulation for a number of genes involved in the methionine cycle generating S-adenosylmethionine (SAM), the universal methyl donor, in inositol transport and catabolism or in trehalose de novo biosynthesis and transport [15]. To further explore the possible roles that these metabolisms have in stress adaptation and symbiosis of *E. aridi*, deletion mutants of regulatory genes involved in the methionine cycle (*nesR*, *sahR*), the trehalose transport and utilization (*thuR*), the inositol catabolism (*iolR*), or in the trehalose denovo biosynthesis (*otsB*) were generated and phenotyped. Finally, to explore the interdependency between regulated pathways, the transcriptional responses of studied genes were assessed in the various mutant backgrounds upon mobilization of vectors that harbored selected promoter fusions to green fluorescence protein (GFP)-encoding gene.

## 2. Materials and Methods

### 2.1. Bacterial Strains and Plasmids

The bacterial strains and plasmids used in this study are listed in Table 1. *Ensifer* strains and derivatives were cultured in tryptone yeast extract (TY) medium [22] at 28 °C. *E. coli* strains were grown at 37 °C in Luria–Bertani (LB) medium [23]. Agar at 2% (*w/v*) was added when needed. For selection of bacterial transformants and transconjugants, sucrose (5%, *w*/*v*) gentamycin (Gm, 10 μg mL^−1^), kanamycin (Km, 50 μg mL^−1^), ampicillin (Ap, 50 μg mL^−1^), rifampicin (Rif, 100 μg mL^−1^), and 5-bromo-4-chloro-3-indolyl-beta-D-galactosidase (X-gal, 80 μg mL^−1^) were added to the media when required.

### 2.2. Mutants’ Constructions

The cloning of *iolR* (PEG374), *thuR* (PEG6269), *nesR* (PEG5735), and *otsB* (PEG4868) 5′ and 3′ regions were performed using a two-step PCR using primers listed in Table 2. The PCR was carried out using as template genomic DNA extracts [29] from 10 mL of LMR001 liquid cultures at the early stationary phase s. First, PCR reactions were performed to amplify separately both the 5′ and the 3′ flanking regions using the proof-reading enzyme Phusion™ (Thermo-Fischer) and recommended chemistry, except for primers (1 µM final concentrations for forward and reverse primers). The cycling conditions included a primary denaturation step of 30 s at 98 °C followed by 35 cycles (98 °C, 10 s/56 °C, 30 s/72 °C, 45 s) and a final elongation step of 7 min at 72 °C. PCR products were purified using Illustra™ GFX PCR DNA and Gel Band Purification kits (GE Healthcare, Chicago, IL, USA), following manufacturer’s recommendations. Then, for each gene to be mutated, a second PCR reaction was set up with purified PCR products from the 5′ and 3′ regions obtained that contain complementary sequences included in the 5′ end of internal primers (see Table 2), thus enabling priming of the amplification. The 25 µL reactions contained 5 µL of 5× GoTaq buffer (Promega), 1.25 µL of MgCl2 (25 mM), 2.5 µL of dNTP mix (2.5 mM each), 0.125 µL GoTaq (5 U/µL), 2 µL of each purified PCR products obtained, and nuclease free water. A primary PCR consisting of a denaturation step of 95 °C for 5 min, followed by 5 cycles (95 °C, 30 s/50 °C, 45 s/72 °C, 50 s) and a final elongation step (5 min at 72 °C) was performed so as to generate chimeric DNA molecules consisting of merged 5′ and 3′ regions. Then, 15 µL of the mix (3 µL of 5× GoTaq buffer, 0.75 µL of MgCl2 (25 mM), 1.5 µL of dNTP mix, 0.125 µL of GoTaq (5 U/µL), 4 µL of external primers (10 µM), and 1.625 µL of nuclease free water) was added to the PCR reaction and subjected to the following cycling conditions: a denaturation step of 95 °C for 5 min followed by 25 cycles (95 °C, 30 s/55 °C, 45 s/72 °C, 50 s) and 5 min at 72 °C. The PCR products were purified from 1% agar gel using Promega Wizard^®^ SV Gel and PCR Clean-Up System, following recommended protocol. As a result of the two amplifications’ process, the PCR-amplified and -purified products were cloned as chimeric fragments into pGEM-T Easy Vector (Promega kit) and transferred into XL2-Blue ultra-competent cells. The resulting positive clones were selected and the plasmids were extracted using the Wizard^®^ Plus SV Minipreps DNA Purification System, digested by the XbaI enzyme, and ligated into suicide vector pJQ200SK [26]. Enzymes were purchased from Promega. The latter plasmid was used to transform *Ensifer aridi* LMR001 cells by the bipartite conjugation method using *E. coli* S17-1 as a donor (Simon et al. 1983) or by tri-parental mating procedure with *E. coli* DH5α [24] as a donor and the helper plasmid pRK2013 [27]. Transconjugants were selected on TY plates containing Rif and 5% sucrose to allow selection of knockout clones that had undergone a double cross-over. Double cross-over gene replacement in mutants was verified by screening for antibiotic-resistant sensibility phenotypes (Rif and Gm, respectively) and also PCR to verify deletion by using specific external and internal primers.

For the construction of the *sahR* (PEG2239) mutant, the 5′ and 3′ regions were first amplified using the primer pairs SAHR-P2239-A-AI/SAHR-P2239-B-PI and SAHR-P2239-C-PI/SAHR-P2239-D-XI (Table 2), respectively. PCR products were then cloned into pGEM-T Easy Vector following manufacturer’s recommendations. Ligations were used to transform competent DH5α cells. The plasmids were extracted and clones containing the inserts oriented from ApaI toward NsiI in the MCS were selected using plasmid and insert specific primers. DNA inserts in purified plasmids were sequenced and further digested with PstI. The clone that contained the 5′ region generated a linearized plasmid that was further dephosphorylated, and the plasmid that contained the 3′ region generated a band corresponding to the linearized vector and a second band that corresponded to the 3′ region cloned that was further extracted from an agarose gel. The latter insert was further ligated into the PstI digested and dephosphorylated vector containing the 5′ region. Ligation mix was used to transform the DH5α cells. A clone containing the 5′ and the 3′ regions in the correct orientation was selected by positive screening using specific primers. Finally, the insert was excised using external restriction sites ApaI and XbaI and cloned into pJQ200SK prior conjugation into LMR001 to generate the Δ*sahR* strain, as described above. Finally, the double mutant Δ*sahR*Δ*nesR* was produced by mutating the Δ*sahR* strain upon double recombination of the pJQ200SK clone containing the *nesR* 5′ and 3′ regions, as described above.

### 2.3. Phenotypic Characterization of the Mutants

#### 2.3.1. Plant Nodulation Assay

Seeds of *Vachellia tortilis* subsp. *raddiana* and endemic Moroccan *Vachellia gummifera* were surface sterilized, germinated, and cultivated, as described previously [15]. Briefly, after germination (5 days in the dark), young seedlings were transferred in pots containing sterilized sand and *Ensifer aridi* strains were inoculated using 1 mL of washed cells at an optical density (600 nm) ≈ 0.7 corresponding to exponential growth phase. Six individual plants were inoculated with each rhizobial strain and sterile water was added for the negative and positive controls. Plants were grown at 26 °C with 16 h day and 8 h night and watered using BD medium for watering [30] supplemented with 3 mM of KNO_3_ for positive controls. Plants were harvested after 6 months of growth, and the nodule number, plant shoot, and root fresh weights were recorded.

#### 2.3.2. Motility, Tolerance to Salt, Detergent, Acid and Oxidative Stresses

The mutants were phenotyped as described previously [15]. Briefly, bacterial motility was calculated by measuring the growth diameter of motile bacteria after 2, 3, or 5 days of incubation at 28 °C in soft TY agar (0.2% *w*/*v*) complemented or not with NaCl at 0.1 or 0.25 M. Salt tolerance was estimated by growing bacteria in TY liquid medium and TY complemented with 0.25, 0.3, or 0.4 M of NaCl and the OD was recorded after 0, 4, 8, 24, 56, 120, 144, 168, and 216 h of growth at 28 °C with shaking. Then, 5 µL of 2-week-old cultures were spotted onto TY agar plates to estimate the long-term survival of bacteria in these different media. Sensitivities of the mutants to oxidative, acid, and detergent stresses were compared to those of the wild-type LMR001 strain. Briefly, bacterial cultures (pre-grown to OD 600 nm ≈ 0.7 in TY liquid medium) were added to 16 mL of soft TY agar containing 0.7% of agar precooled to 45 °C and poured onto TY agar plates. Once solidified, a paper disc (6 mm diameter) soaked with 5 µL of H_2_O_2_ (2 M), HCl (5.5 M), or SDS (10% *w*/*v*) was placed onto the surface of the plates that were incubated at 28 °C for three days prior to recording the diameters of the inhibition zones. The means of the three replicates were calculated.

### 2.4. Transcirptional Fusion Assays

The promoters of selected genes were cloned previously in the broad-host-range mobilizable vector pROBE-NT and studied in *E. aridi* LMR001 and Δ*rpoE2* strains [15]. These included the promoter regions of methionine cycle regulatory genes *nesR* (PEG5735) and *sahR* (PEG2239); GSR regulatory genes *rsiA1* (PEG2540) and *rsiB1* (PEG2541); genes involved in the trehalose import system and utilization *thuEFGKAB* (PEG6268-PEG6262) and its regulator *thuR* (PEG6269) or its endogenous biosynthesis *otsBA* (PEG4868, PEG4869) and *treZ* (PEG5323); and, finally, genes involved in inositol catabolism *iolC* (PEG373) and its regulation *iolR* (PEG374). Here, to explore the mutations’ effects on the transcriptional regulation of these genes, pPROBE derivatives were transferred by biparental conjugation into the new mutants using S17-1 *E. coli* as a donor. To estimate the relative expression of targeted genes’ transcriptions, the median relative expression of the 11 primary measures obtained every half hour was calculated for each mutant upon correction to controls and normalization by OD (600 nm) as previously described [15] and the data obtained from two kinetics were averaged.

## 3. Results and Discussion

**Figure 1:**
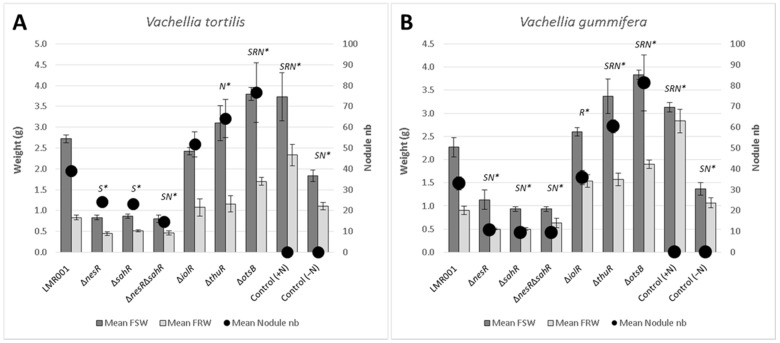
Effects of the mutations on symbiosis development with *Vachellia*. The mean fresh shoot and root weights (shown, respectively, as FSW, dark grey and FRW, light grey; left axis) and nodule numbers (filled black circles; right axis) in *V. tortilis* (**A**) and *V. gummifera* (**B**) are shown for the mutants and non-inoculated control plants either watered using nitrogen complemented nutrient solution (+N) or without nitrogen (−N), as indicated at the bottom of the bar graphs. Error bars corresponding to standard deviations and significant differences with LMR001 data (*p*-value < 0.05, Tukey’s Test) are indicated by “*” preceded by the letter “S”, “R”, and/or “N” for mean shoot, root weights, and nodule numbers, respectively.

To know whether the mutated genes were important for symbiosis development, the mutants were inoculated on two compatible hosts *V. gummifera* and *V. tortilis*, and their growth and nodulation were compared to those obtained with the LMR001 wild-type strain (Figure 1). 

**Figure 2:**
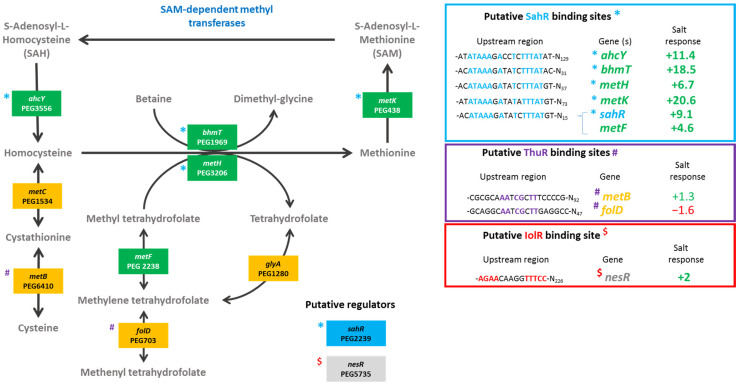
Metabolism of methionine. *Ensifer aridi* predicted encoding genes (PEGs) involved in the metabolism of methionine are shown in the metabolic pathway. The presence of putative SahR, ThuR, and IolR binding sites upstream of genes (shown in the right boxes) are indicated, respectively, by “*”, “#”, and “$” on the left of genes when identified, and the position of conserved residues are bolded and colored accordingly in boxes. The salt response refers to fold change previously found by RNAseq [15]. Positive fold changes indicating upregulation (green) and negative ones showing repression (red) in salt containing medium are bolded when above 2.

We found that deletions of genes involved in the regulation of the methionine cycle (*nesR* and *sahR*) significantly reduced the plant shoot weights for both *Vachellia* species as compared to the wild-type strain. Furthermore, the nodulation was also significantly lower than that of the LMR001 strain when the two regulatory genes were mutated in *V. tortilis*. In *V. gummifera*, all three mutants (*ΔnesR*, *ΔsahR*, and the double mutant *ΔnesRΔsahR*) significantly reduced the nodule number, suggesting that methionine cycling regulation generating the universal methyl donor SAM is important for proper symbiosis development and functioning. The regulation of the methionine cycle is complex and involves several transcription factors, such as NesR and SahR or RNA regulatory elements (SAM riboswitch) that remain to be investigated in *Ensifer aridi*. Nevertheless, given the strong homologies with the LuxR solo NesR of *S. meliloti* [31] and the ArsR family repressor SahR found in diverse proteobacteria, including rhizobiales [32], for which a conserved DNA-binding domain could be identified upstream LMR001 *sahR*, *ahcY*, *metH*, *bhmT*, and *metK* (Figure 2), our data suggest that a fine-tuning of the SAM-generating methionine cycle is essential for symbiosis establishment and functioning. Interestingly, it was recently shown in *S. meliloti* that the SAM synthetase MetK was also binding stress-inducible RNAs which previously showed to interact with the RNA chaperone Hfq and, more surprisingly, other mRNAs [33] which suggests that MetK-mediated post-transcriptional regulation may occur not only through epigenetics via the production of the universal methyl donor SAM but also by interacting with various RNAs and mRNAs.

**Figure 3:**
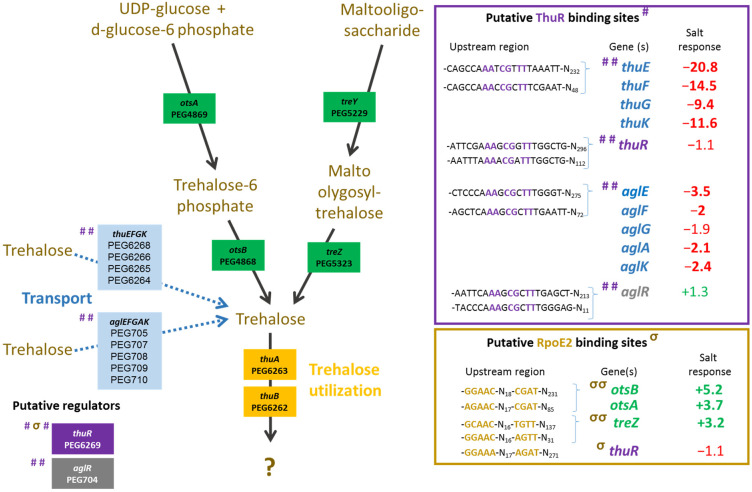
Metabolism of trehalose. *Ensifer aridi* predicted encoding genes (PEGs) involved in the metabolism and transport of trehalose are shown in the metabolic pathway. The presence of putative ThuR and RpoE2 binding sites upstream of genes (shown in the right boxes) are indicated by “#” and “σ”, respectively, on the left of genes when identified, and the position of conserved residues are bolded and colored accordingly in boxes. The salt response refers to fold change previously found by RNAseq [15]. Positive fold changes indicating upregulation (green) and negative ones showing repression (red) in salt containing medium are bolded when above 2.

The plant test also showed that a mutation in *thuR* or *otsB*, involved in the trehalose transport and/or metabolism, stimulated nodulation with both of the tested plants, while roots and shoots were also significantly increased in *V. gummifera*. Because ThuR is a LacI-GalR family regulator predicted to repress transport and catabolism genes (*thuEFGKAB*) through binding to upstream conserved motifs [34] also identified in *E. aridi* (Figure 3), the *thuR* deletion is expected to increase trehalose uptake capacity and utilization which may improve intracellular fitness and, thus, symbiotic efficiency; however, it remains to be functionally demonstrated. Strikingly, the *otsB* mutation significantly improved both plant weight and nodule number with the two plants. The trehalose-6P phosphatase OtsB is involved in the second step of the trehalose biosynthesis pathway which utilizes glucose-6P and UDP-glucose as primary substrates (Figure 3). A deletion of this gene should, therefore, result in an accumulation of trehalose-6P in the cell. Interestingly, an increased trehalose-6P synthase production by genetically manipulated rhizobial strains was reported to increase nodulation and plant biomasses in *Phaseolus vulagris* [35] or chickpea upon salt stress [36]. Even though much remains to be done to characterize factors involved in trehalose-6P-mediated plant growth and stress tolerance improvements, this phosphosugar is gaining increasing interest. It was recently shown to not only act as a compatible solute but also as signaling molecule in plant notably by modulating SnRK1 phosphorylation status, a central protein kinase involved in the coordination of the plant development that can lead to improved growth, notably under stress conditions [37,38]. Additional work is required to determine whether trehalose-6P accumulates in the Δ*otsB* strain during symbiosis development and functioning and whether the effects are linked to improved fitness and/or signaling.

**Figure 4:**
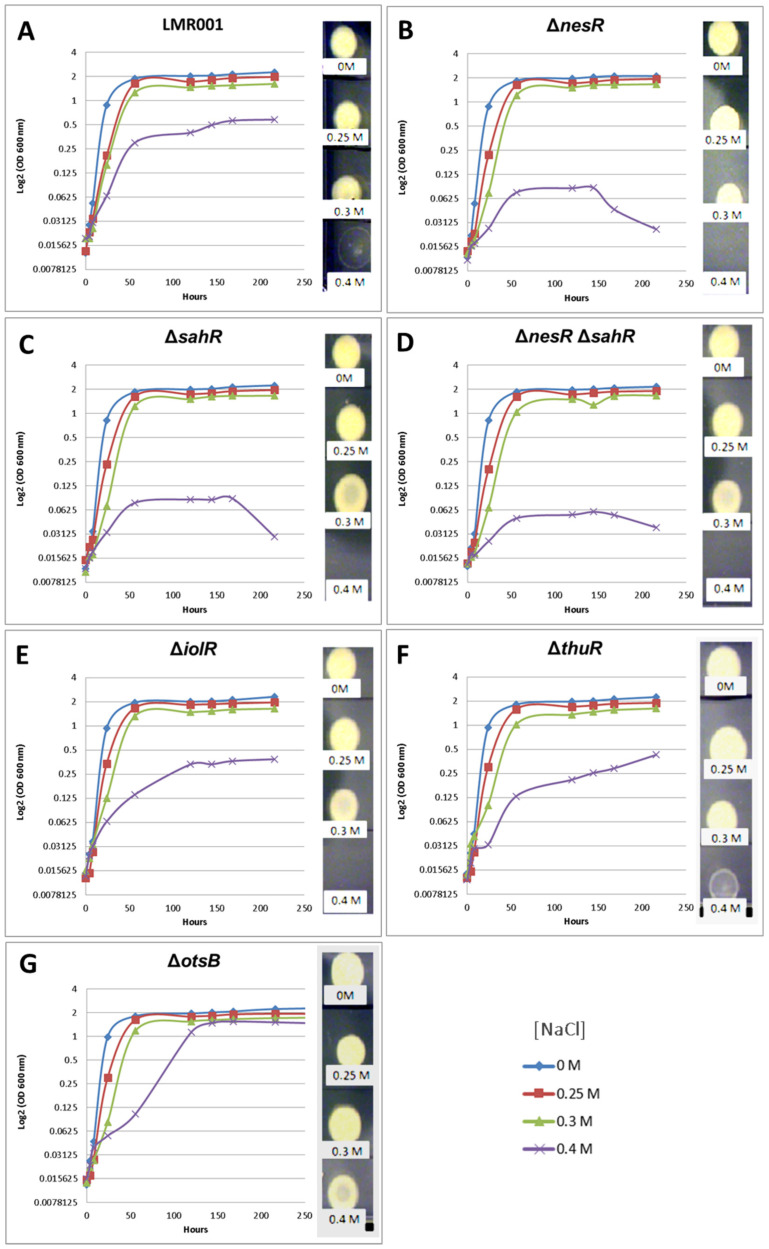
Effects of the mutations on salt tolerance. The growth kinetics are shown for the LMR001 (**A**), the strains Δ*nesR* (**B**), Δ*sahR* (**C**), Δ*nesR*Δ*sahR* (**D**), Δ*iolR* (**E**), Δt*huR* (**F**), and Δ*otsB* (**G**) in TY complemented with 0, 0.25, 0.3, or 0.4 M of NaCl (color code indicated at the bottom right of the figure). The OD (600 nm) corresponding to the mean of three replicates is shown using log 2 scale up to 9 days of growth (line graphs), and 5 µL of 2-week-old cultures was spotted onto TY to estimate long-term survival (pictures shown on the right of each line graph).

The tolerance of the mutants to salt-induced osmotic upshift was compared to that of the LMR001 strain (Figure 4). The growth kinetics of tested strains in the TY medium, or when it was supplemented with 0.25 or 0.3 M of NaCl, were very similar. However, in the medium containing 0.4 M of salt, the growth of regulator mutants Δ*nesR*, Δ*sahR*, and the double mutant Δ*nesR*Δ*sahR* were strongly impaired, suggesting that a fine-tuning regulation of the methionine cycle is important for salt tolerance in *Ensifer aridi*. The deletion of the trehalose uptake and utilization regulator *thuR* resulted in a continuous growth in the medium containing the highest salt concentration. Interestingly, while the otsB mutation resulted in reduced growth as compared to LMR001 in TY containing 0.4 M of salt after 2 days, the mutation enabled the bacterium to reach a higher OD at the stationary phase which almost reached the density observed in other media, suggesting that the strain impaired in trehalose phosphate phosphatase activity improves salt tolerance in LMR001. To estimate the viability of bacteria in tested media, five microliters of 2-week-old cultures were spotted onto the TY agar medium. While strains were all able to survive in media containing 0.3 M of NaCl or less, different growth phenotypes were found when bacteria were grown in the medium containing the highest salt concentration.

We found that after 2 weeks of growth in the medium supplemented with NaCl at 0.4 M, the LMR001 strain was still alive even though it showed a weak growth, as compared to cells grown in other media. All three mutants involved in methionine cycle regulation did not survive, showing that, at a higher osmotic strength, the proper regulation of the methionine cycling is essential. The Δ*iolR* strain that reached a lower OD at the stationary phase as compared to LMR001 strain did not survive prolonged incubation in TY supplemented with 0.4 M of NaCl. Interestingly, a deletion of *thuR* and more importantly *otsB* resulted in an improved long-term survival in the high salt concentrated medium as compared to the LMR001 strain. These results are globally in line with plant tests and further support the important role of stress-responsive genes in symbiosis functioning [39].

**Figure 5:**
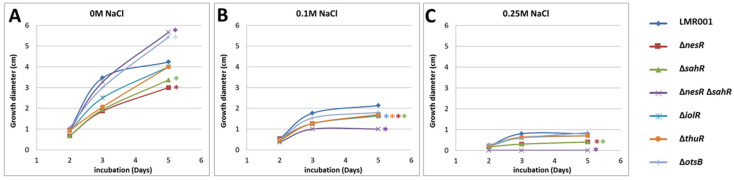
Effects of the mutations and osmotic strength on bacterial swimming. The motility was estimated by measuring the growth diameter from triplicate TY soft agar plates supplemented with salt 0 M (**A**), 0.1 M (**B**) or 0.25 M (**C**). The mean motility after 2, 3, and 5 days are shown for the LMR001 and the 6 mutants (indicated on the right of the figure). Significant differences with LMR001 motility after 5 days (*p*-value < 0.05, Tukey’s Test) are indicated by “*” on the right of lines using the same color code.

The motility of *Ensifer aridi* was previously shown to decrease in the presence of salt in the medium [15]. To test whether the mutated genes modified the swimming capacity of LMR001, their motility was compared to that of the LMR001 strain in soft agar plates that contained either TY medium or TY complemented with 0.1 or 0.25 M of NaCl. Figure 5 shows the results of the swimming assay for all strains. As shown for the LMR001 strain, the presence of salt in the medium reduced the motility in a dose-dependent manner. However, we found that after 5 days of growth in the TY medium, the Δ*otsB* showed continuous motility that exceeds that of the LMR001 strain. Whether this phenotype results from an increased trehalose-6P remains to be addressed. A similar phenotype was found for the double mutant Δ*nesR*Δ*sahR* when, in contrast, single mutants of the methionine cycle regulators Δ*nesR* and Δ*sahR* presented a lower motility. These results are surprising as we would expect that the double mutation should result in a stronger effect. When salt was added in the medium, the motility was more strongly reduced for the double mutant as compared to the LMR001 strain and the single mutants and even totally impaired in the medium containing 0.25 M of salt, showing the importance for the bacterium to finely tune the methionine cycle for proper functioning. Given the involvement of methyl-accepting chemotaxis proteins in the regulation of diverse cellular activities including flagellum biosynthesis, it is not surprising that an alteration of the SAM-generating methionine salvage pathway regulation generates motility phenotypes. Nevertheless, additional work is required to characterize the effects of the mutations on SAM and whether these are responsible for the observed motility effects. Finally, after 5 days of growth, the Δ*iolR* and the Δ*thuR* mutants showed significantly lower motility in the medium containing 0.1 M of NaCl as compared to the LMR001 strain, suggesting that both trehalose and inositol affect swimming; however, mechanisms underlying these effects remain to be determined.

**Figure 6:**
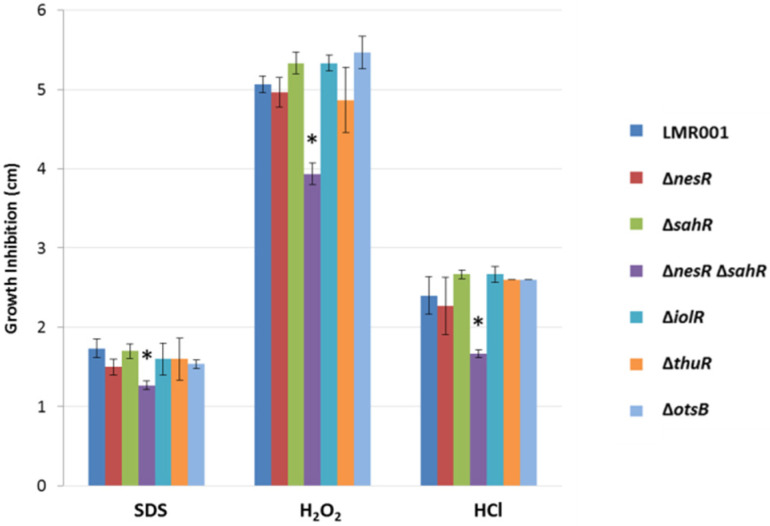
Effects of the mutations on detergent, oxidative and acids stresses. The mean diameters of the growth inhibition zone are shown for the 6 mutants (indicated on the right of the figure). The size of the inhibition zone is inversely correlated to the tolerance as the chemicals (indicted at the bottom of the graph) diffuse away from the disc into the agar (see Section 2). Error bars correspond to standard deviations and significant differences with LMR001 data (*p*-value < 0.05, Tukey’s Test) are indicated by “*” above the corresponding bar.

The tolerance to detergent (SDS), oxidative (H_2_O_2_), and acid (HCl) stresses of the studied strains was evaluated by comparing the growth inhibition zone resulting from their diffusion from a disc soaked with these chemicals (see Section 2). We found that only a double mutation of the two methionine cycle regulators resulted in a significant difference when compared to the LMR001 strain (Figure 6). The reduced inhibition zone found in the double mutant suggests that its membrane structure is altered, resulting in improved resistance to the three stresses which require additional work to be characterized.

**Figure 7:**
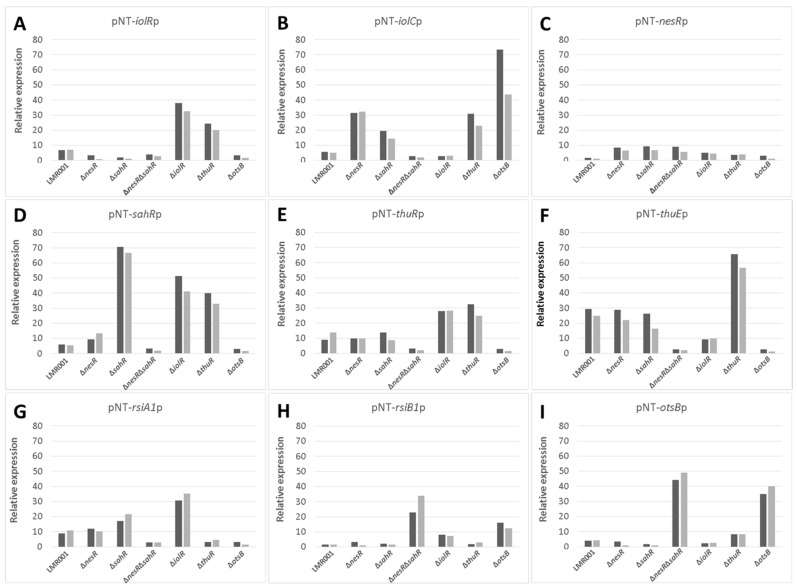
Effects of the mutations on the relative expression level of selected genes using promoter–gfp fusion reporter system. The relative expression of iolR (**A**), iolC (**B**), nesR (**C**), sahR (**D**), thuR (**E**), thuE (**F**), rsiA1 (**G**), rsiB1 (**H**), and otsB (**I**) in the studied strains (indicated at the bottom of each bar graph) in TY or TY complemented with salt (250 mM of NaCl) are shown as dark and light grey bars, respectively. The mean relative expressions were calculated using the median fluorescence of 11 measures recorded every half an hour that were further corrected to controls and normalized to the OD (600 nm).

**Figure 8:**
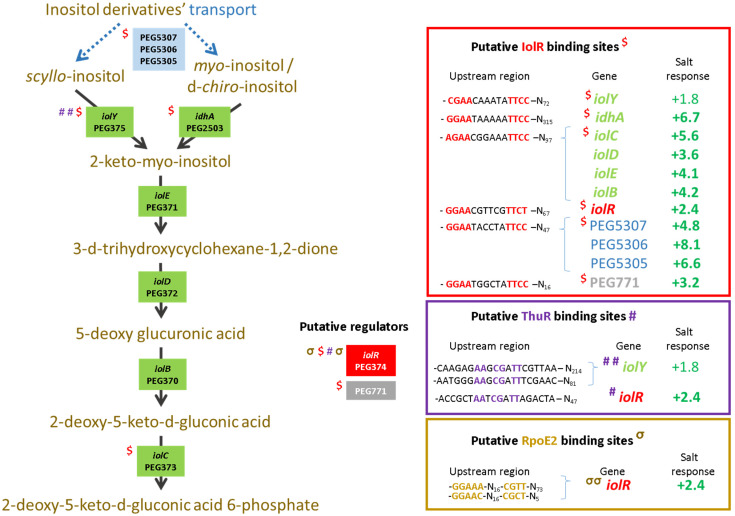
Catabolism of inositol. *Ensifer aridi*-predicted encoding genes (PEGs) involved in the transport and catabolism of inositol are shown in the metabolic pathway. The presence of putative IolR, ThuR, and RpoE2 binding sites upstream of genes (shown on the right boxes) are indicated, respectively, by “$”, “#”, and “σ” on the left of genes when identified and the position of conserved residues are bolded and colored accordingly in boxes. The salt response refers to fold change previously found by RNAseq [15]. Positive fold changes indicating upregulation (green) in salt containing medium are bolded when ≥ 2.

To further describe the effects that mutations had on the transcriptional control of the inositol, the methionine, and the trehalose metabolisms, the relative expression levels of selected genes were assessed using the pPROBE derivatives previously screened in the Δ*rpoE2* strain [15]. Figure 7 shows the mean relative expression obtained for all mutants. We found that in the Δ*iolR* and Δ*thuR* mutant backgrounds, *iolR* promoter activity was higher than in the wild-type strain, while in other mutants, the relative expression remained low and close to the background level (Figure 7A). The regulator IolR is a RpiR-like repressor shown to be involved in the regulation of the inositol catabolism in *S. meliloti* [40,41]. A regulatory model was proposed which involves IolR binding to promoter regions of target genes that are released when the intermediary product of the inositol catabolism 2-deoxy-5-keto-D-gluconic acid 6-phosphate (DKGP) is produced [41]. The promoters of genes involved in studied pathways of *E. aridi* were screened for putative regulator binding sites identified by Kohler and colleagues [41]. Interestingly, we found putative binding sites upstream of *idhA*, *iolY*, *iolR*, and *iolC* in LMR001 (Figure 8), suggesting a similar regulation in *Ensifer aridi* as all these genes were previously found to be induced upon salt stress [15] (Figure 8). In line with this model, we found that the mutation of *iolR* resulted in an induction of its own transcription, supporting a feedback regulatory loop (Figure 7A). However, we found that the *iolC* transcription was not induced in the *iolR* mutant background (Figure 7B), indicating that IolR is necessary for proper *iolC* transcription, thus suggesting a complex regulation of the inositol catabolism that may involve additional factors for proper regulation. Nevertheless, it is also possible that the *iolR* deletion resulted in an unexpected modification of the downstream genes involved in inositol catabolism that could impair the production of the product effector KDGP suggested to be required for antagonizing IolR repression. It should be noted that Kohler and colleagues [41] showed that even though IolR binding domains were found upstream of the *iolY*, *iolR*, and *iolC* genes, all the *iolYRCDEB* genes were transcribed as a single mRNA forming an operon. Recently, a *iolR* mutant in *Aeromonas* was shown to act both as a repressor and as an activator for a high number of genes [42]. Interestingly, a sequence close to the consensus IolR binding site was found in the intergenic region of the *rsiA1-rsiB1* genes involved in the GSR (Figure S1). This putative IolR binding site overlaps with the predicted RpoE2-binding motif [20,43] located upstream of *rsiB1* (Figure S1) which may suggest a possible implication of the IolR repressor in the modulation of the GSR. It should be noted that two motifs close to the consensus sequence of the ecfG sigma factor RpoE2-binding site were also predicted upstream of the *iolR* gene and the IolR putative binding site overlapping the primary putative RpoE2 binding site (Figure 8), which would support a possible cross regulation. By screening the genome of *E. aridi* for the presence of additional putative IolR binding sites, we found several consensus sequences. Among them, we identified the sequence motif upstream of a predicted ABC-type inositol transport system (PEG5307-5306-5305) that was also found induced by salt stress [15] (Figure 8). 

Furthermore, another putative IolR binding site was also found upstream of PEG771, a LacI-type transcriptional repressor predicted to control inositol transport and utilization in *R. etli* [34]. Interestingly, a regulatory motif predicted as a binding motif of *R. etli* IolR (RHE_CH02362) by Regprecise was found twice in the intergenic region of PEG771–772, suggesting that inositol metabolism and transport regulation involves additional genes in *E. aridi* that may be modulated by IolR. Among putative IolR binding sites, we also found a putative IolR binding site upstream of the *nesR* start codon (Figure 2) which could suggest IolR-dependent methionine cycle regulation. However, this motif was located within the 5′ end of the *pip* gene, and *nesR* transcription using the GFP reporter system was close to background levels which make it difficult to assess. We also found that the *iolC* transcription was higher in the strains Δ*nesR*, Δ*sahR*, Δ*thuR*, and Δ*otsB*, while its transcription was close to background levels in the double mutant Δ*nesR*Δ*sahR* (Figure 7B). Whether these increased *iolC* transcriptions are directly linked to the methionine and trehalose cycles remains to be determined, but these data strongly support a complex regulation of these metabolisms that appear interdependent and additional work is required to test the targets of IolR that may not be restricted to the regulation of inositol catabolism.

The relative expression of *nesR* encoding a LuxR solo showed to positively regulate the methionine salvage pathway generating SAM in *S. meliloti* [31], was higher in Δ*nesR*, Δ*sahR* and the double mutant Δ*nesR*Δ*sahR* as compared to the other strains (Figure 7C). However, the expression remained low which makes it difficult to interpret. In contrast, the alphaproteobacterial ARS-type regulator SahR, which was predicted to act as a repressor [32], showed contrasting relative expression levels in the various strains (Figure 7D). Novichkov and colleagues showed that, in *Desulfovibrio*, SahR binds to operators of genes involved in SAM cycling depending on the cellular level of the effector S-adenosylhomocysteine (SAH) [32]. Here, the highest *sahR* relative expression was found in the Δ*sahR* strain supporting autoregulation. Accordingly, the promoter region of *sahR* in LMR001 possessed the predicted binding motif conserved among rhizobia that could also be identified upstream of the known target genes *ahcY*, *metH*, *metK*, and *bhmT* [32] (Figure 2). Given the fact that all these genes were found upregulated by salt previously [15], it is tempting to speculate on the role of SahR as a repressor of the SAM cycling in *E. aridi*. This raises the question regarding the regulatory mechanisms of this important pathway that, once again, involves several regulators, which is nevertheless not surprising given the importance of SAM as a universal methyl donor and the downstream activities it is involved in for cell functioning. Screening of the LMR001 genome for the presence of other putative SahR binding sites did not return any other hit, suggesting that SahR is primarily involved in SAM cycling regulation. However, the *sahR* promoter activity appeared also increased in the Δ*iolR* and the Δ*thuR* strains, as previously shown in Δ*rpoE2* [15], which further show the pleiotropic effects alterations of the methionine cycling have on the other metabolisms.

Regarding the relative expression of *thuR* (Figure 7E) and *thuE* (Figure 7F), we previously found that *thuR* transcription was reduced in the Δ*rpoE2* strain in contrast to that of *thuE* which supported a function of ThuR as a repressor of its own transcription and that of the *thuEFGKAB* operon that possesses 2 predicted ThuR binding motifs in their promoter regions (Figure 3) [15,34]. Here, we found that the *thuR* mutation resulted in inductions of both *thuR* and *thuE*, while the induction of *thuR* in the Δ*iolR* strain showed a concomitant repression of *thuE* when compared to LMR001. However, a strong repression of *thuR* in the Δ*otsB* and the double mutant Δ*nesR*Δ*sahR* was found, which did not result in *thuE* inductions. Nevertheless, as shown in other LacI type regulators, such as the close regulator AglR, the DNA binding of such type of transcription factors depends on the presence of specific sugars as effectors whose concentrations and availability may be altered in the later strains. Indeed, given the inductility of *thuEFGK* by trehalose [44], it appears probable that the trehalose is involved in ThuR binding affinity to target promoters and a mutation of the trehalose-6P phosphatase may alter the availability of the effector to the repressor. Furthermore, as shown above, the double mutant Δ*nesR*Δ*sahR* presented a stronger tolerance to detergent, oxidative, and acid stresses, suggesting a modification of the cell wall in this mutant that may modify cellular exchanges that could alter the availability of effectors to the regulator. However, additional work is required to further characterize the regulation of *thu* genes. It is interesting to note the presence of a motif (GGAAA-N17-AGAT) that resembles the RpoE2 sigma factor binding domains between these two ThuR recognition motifs upstream of the *thuR* start codon which would suggest that *thuR* transcription is RpoE2-mediated, in accordance with *thuR* transcriptional repression reported in the Δ*rpoE2* strain [15]. The genome of the LMR001 strain was also screened for additional putative ThuR binding sites [34]. Among the conserved residues that matched LMR001 intergenic regions, putative binding motifs were found upstream of *folD* and *metC* involved in the methionine metabolism (Figure 2). Interestingly, in Δ*thuR*, *sahR* transcription was induced, which was shown to be activated through interaction of the SAH, i.e., the intermediary product of the methionine cycle acting as SahR effector [32], supporting the implication of ThuR in the methionine cycling. Nevertheless, whether ThuR is directly involved in the regulation of methionine precursors’ homeostasis remains to be determined experimentally. In addition, we also found the putative ThuR binding motif once upstream of the *iolR* gene and twice before the *iolY* start codon (Figure 8), which supports the involvement of ThuR in the regulation of the inositol catabolism. Again, *iolR* transcription was also found activated in Δ*thuR*, further supporting interplay between regulators of the methionine and trehalose metabolisms. 

Among the putative ThuR binding sites identified, we also found the presence of two motifs upstream of the PEP carboxykinase-encoding gene *pcKA* (PEG3563) that was also strongly repressed by salt [15]. Interestingly, Di Cenzo and colleagues [45] showed that, in *S. meliloti*, *pckA* was regulated by PckR, another LacI-type transcriptional regulator that binds to a motif containing the following conserved residues (5′-TNNAANCGNTT-3′) that overlaps the predicted ThuR binding motif. Previous experimental data suggested a complex regulatory mechanism that relied on the presence of a single or a double PckR binding domain in the target promoter sequences which would result, respectively, in an induction or a repression of regulated genes in the presence of PEP, and inversely in the absence of the effector [45]. PckR was shown to regulate several genes of the glycolytic and gluconeogenic pathways, among which *pckA* and *fbaB* that possessed two PckR binding domains, and *zwf*, *mgsA*, and *eda2*, which possessed a single PckR binding domain, that were inversely regulated as compared to *pckA* and *fbaB*. Similarly, in *E. aridi*, both the presence and the number of PckR (PEG3052 in LMR001) binding domains could be identified in the promoter regions of these genes and similar expression profiles were found. Indeed, we found previously that a salt stress resulted in a strong repression of both *pckA* (PEG3563) and *fbaB* (PEG2954) and an induction of *zwf* (PEG714), *mgsA* (PEG118), and *eda2* (PEG3069) [15]. This corroborates Di Cenzo and colleagues’ work on *S. meliloti* and suggests that, according to their regulatory model, under salt stress, *E. aridi* cells contain high levels of PEP. It should be noted that one of the two predicted ThuR binding sites upstream of *thuR* and *aglE* contained the PckR conserved motif, suggesting a possible involvement of PckR in ThuR-mediated regulation. di Cenzo and colleagues proposed several scenarios, including one that was based on a displacement of another negative regulator, thus enabling RNA polymerase to access the promoter. Given the fact that the predicted PckR binding motif includes conserved nucleotides of the ThuR binding motif, the ThuR regulator could be involved in modulating PckR-regulated genes; however, much work remains to be done to further characterize this complex regulatory mechanism and a possible interplay.

The transcriptional regulation of *rsiA1* and *rsiB1* was also studied in the various LMR001 derivatives (Figure 7G and Figure 7H, respectively). Regarding the *rsiA1* relative expression, we found that, in comparison to the LMR001 strain, three mutants (Δ*nesR*Δ*sahR*, Δ*thuR*, and Δ*otsB*) presented a lower expression. However, Δ*sahR* and, more importantly, Δ*iolR* presented a higher relative expression. Regarding the *rsiB1* expression, three mutants (Δ*nesR*Δ*sahR*, Δ*iolR*, and Δ*otsB*) presented an increased transcription level as compared with LMR001. It is interesting to note that both *rsiA1* and *rsiB1* relative expressions were increased in the Δ*iolR* strain as compared to the WT. Even if additional experimental data are required, the presence of a putative IolR binding site located in their promoter regions may be involved in modulating their transcriptional regulation and supports a role of IolR in GSR tuning. Surprisingly, both Δ*nesR*Δ*sahR* and Δ*otsB* strains presented a reduced expression of *rsiA1*, while we observed stronger expressions of *rsiB1* as compared to the WT strain. Because the anti-anti-sigma factor RsiB1 promotes RpoE2 recruitment to its operators through competitive binding to the anti-sigma factor RsiA1, our results suggest that GSR regulation may be altered in these mutants. Nevertheless, *rpoE2* is co-transcribed with *rsiA1* and the GSR is finely tuned and requires RsiB1 phosphorylation through a phosphorylation cascade that is not yet fully characterized, and studies are needed to functionally demonstrate the effects that these mutations may have on GSR. Interestingly, both strains presented a strong induction of the RpoE2-mediated *otsBA* operon (Figure 7I), suggesting that GSR was activated in these mutants. Surprisingly, with the exception of the *iolC* gene that was highly induced upon *otsB* deletion, the relative expression profiles obtained for Δ*nesR*Δ*sahR* and Δ*otsB* strains were highly similar which contrasts with their responses to stress or their symbiotic performances. The fact that *otsB* deletion resulted in an induction of the *otsBA* operon suggests that, in this mutant, the cells accumulate the phosphor-sugar trehalose-6P as the phosphatase OtsB is nonfunctional. Whether tolerance to salt and symbiosis efficiency improvements are due to higher levels of trehalose-6P in the Δ*otsB* strain remains to be determined but further supports the idea that trehalose and its phosphorylated derivative are key metabolites for stress adaptation and plant growth promotion notably under abiotic stress [46]. Interestingly, Sengupta and colleagues previously showed in yeast that trehalose phosphate synthase methylation by a Cystein methyl transferase resulted in increased trehalose-6 phosphate biosynthesis [47], further supporting possible interplay between SAM-generating methionine cycling and trehalose metabolism. Additional research is, however, necessary to test whether such a type of post-translational regulation also occurs in bacteria.

## 4. Conclusions

In order to decipher the complex response of *E. aridi* to an osmotic upshift, a previous study based on RNA-seq enabled the identification of several metabolisms that were altered by salt. Despite activation of regulatory genes involved in the GSR, a deletion of the extra cytoplasmic function *rpoE2* sigma factor-encoding gene did not result in phenotypic alterations, which suggested that alternative regulatory mechanisms could be recruited [15]. Here, a series of mutants in genes involved in the metabolisms of trehalose (*thuR*, *otsB*), inositol (*iolR*), and SAM-generating methionine (*nesR* and *sahR*) that appeared regulated upon salt stress were produced and phenotyped. To further describe the possible regulatory circuits between these metabolisms, the transcriptional state of selected genes and notable regulators involved in these metabolisms were assessed in these mutant backgrounds using a promoter—*gfp* fusion-based reporter system. The results show that mutations in genes involved in the methionine cycling regulation altered the symbiosis development and functioning with two compatible hosts, suggesting that a fine-tuned regulation of the methionine, and possibly SAM, is required. These mutants also showed a reduced growth in a medium containing 0.4 M of NaCl with a motility that was more strongly inhibited by salt than that of the wild-type LMR001 strain which may reduce plant colonization and in planta rhizobial fitness. Surprisingly, when the two genes *nesR* and *sahR* were deleted, the strain appeared more tolerant to detergent, oxidative, or acid stresses, suggesting an alteration of the cellular membranes. In contrast, mutants in the *thuR* or *otsB* genes resulted in improved symbiosis, showing that a modulation of the trehalose metabolism represents an interesting way to promote symbiosis and, thus, plant growth which was also correlated with an improved growth in a medium containing 0.4 M of NaCl. The transcription of genes encoding repressors IolR, ThuR, and SahR were induced when they were deleted, suggesting autoregulation which was shown in other bacteria for IolR and SahR and predicted for ThuR. We also found that IolR was required for proper *iolC* induction but was, in contrast, induced in Δ*nesR*, Δ*sahR*, Δ*thuR,* and Δ*otsB,* suggesting that the regulation of trehalose and methionine metabolisms can alter inositol catabolism while necessitating additional experimental evidences. Nevertheless, if *nesR* promoter activity remained low in all mutant backgrounds, we also found that the transcriptional state of the putative SahR repressor was also induced upon *thuR* or *iolR* mutations which further demonstrates an interplay between the studied metabolisms’ regulations. We also found that deletion of the *iolR* regulatory gene altered *thuR* and *thuE* transcriptions. When the transcriptional regulation of the *rsiA1* and *rsiB1* genes was assessed in the studied strains, different profiles were found depending on the mutant. Indeed, the *thuR* deletion resulted in a lower transcription of *rsiA1* that was also found in the Δ*otsB* and Δ*nesR*Δ*sahR* strains. In contrast, the two later strains presented a strong induction of the *rsiB1* gene, suggesting that a deregulation of the methionine and trehalose metabolisms alter the GSR. Finally, GSR may also be influenced by inositol, as both *rsiA1* and *rsiB1* genes were induced in the Δ*iolR* strain. Whether these regulations are under the direct control of studied transcriptional regulators that possessed, in some cases, several putative binding motifs in their promoter regions, or indirect control through the involvement of yet unknown factors, may now be further explored. Nevertheless, the present study clearly shows that the stress-responsive genes are regulated by complex regulatory circuits, suggesting an interplay of genes involved in methionine, inositol, and trehalose regulatory pathways.

## Figures and Tables

**Table 1:** List of bacteria and plasmid used in the study.

Strain/Plasmid Name	Description	Source
Bacterial strains		
LMR001^T^	*Ensifer aridi* LMR001^T^ isolated from *Vachellia gummifera* grown in Merzouga desert sand dune	[4]
LMR001∆*nesR*	*nesR* deletion mutant of LMR001	This work
LMR001∆*sahR*	*sahR* deletion mutant of LMR001	This work
LMR001∆*nesR*∆*sahR*	*nesR* deletion mutant of LMR001∆*sahR*	This work
LMR001∆*iolR*	*iolR* deletion mutant of LMR001	This work
LMR001∆*thuR*	*thuR* deletion mutant of LMR001	This work
LMR001∆*otsB*	*otsB* deletion mutant of LMR001	This work
*E. coli* DH5α	*hsdR*17 *endA1 thi*-1 *gyrA*96 *relA1 recA1 supE*44 DlacU169 (f80*lacZ*DM15)	[24]
*E. coli* S17-1	*recA* [SmR], *thi,* pro, RP4-2-Tc:Mu: *aph:*:Tn7λpir.	[25]
*E. coli* XL2 Blue Ultra-competent	*endA1 supE*44 thi-1 *hsdR*17 *recA1 gyrA*96 *relA1 lac* [F’ *proAB lacI*qZΔM15 Tn10 (TetR) Amy CamR]	Stratagene
*Plasmids*		
pJQ200SK	pACYC184-derived (p15A) suicide vector (GmR)	[26]
pRK2013	Helper plasmid containing the ColE1 replicon with RK2 *tra* genes (KmR)	[27]
pGEM-T Easy	pUC origin, Multi Cloning Sites (MCS), *lacZ* gene fusion, β-lactamase coding region, *lac* operon sequences (ApR)	Promega
pBluescript KS(+)	ColEI, F1 Origin, MCS, lacZ gene fusion, β-lactamase coding region, lac operon sequences (ApR)	Stratagene
TOPO vecteur	pUC origin, région *Plac* promoteur, *lacZ* α-*ccdB* gene fuision. Topoisomerase enzyme (KmR, ApR)	Invitrogen
pPROBE-NT	pVS1-derived (p15a) vector, *gfp* (GmR)	[28]
pNT-*sahR*p	pPROBE NT containing *sahR* (PEG2239) promoter fused to *gfp* (KmR)	[15]
pNT-*nesR*p	pPROBE NT containing *nesR* (PEG5735) promoter fused to *gfp* (KmR)	[15]
pNT-*rsiA1*p	pPROBE NT containing *rsiA1* (PEG2540) promoter fused to *gfp* (KmR)	[15]
pNT-*rsiB1*p	pPROBE NT containing *rsiB1* (PEG2541) promoter fused to *gfp* (KmR)	[15]
pNT-*thuE*p	pPROBE NT containing *thuE* (PEG6268) promoter fused to *gfp* (KmR)	[15]
pNT-*otsB*p	pPROBE NT containing *otsB* (PEG4868) promoter fused to *gfp* (KmR)	[15]
pNT-*thuR*p	pPROBE NT containing *thuR* (PEG6269) promoter fused to *gfp* (KmR)	[15]
pNT-*iolC*p	pPROBE NT containing *iolC* (PEG373) promoter fused to *gfp* (KmR)	[15]
pNT-*iolR*p	pPROBE NT containing *iolR* (PEG374) promoter fused to *gfp* (KmR)	[15]

**Table 2:** Primers used in this study.

Target Gene (bp)	Primer *	Sequence (5′-3′) *	5′ Region Size (bp)/Distance to Start (nt)	3′ Region Size (bp)/Distance to Stop (nt)	Deletion (bp)
*iolR* (861)	IOLR-P374-A-**XI**	CC**TCTAGA**CGAGATTTACGGCTCCAAGG	457/−10		689
	IOLR-P374-B-**HIII**	TCAAAGACACCAC**AAGCTT**CTCCAGCTTGCTCGTGTTC			
	IOLR-P374-C-**HIII**	**AAGCTT**GTGGTGTCTTTGACCGACTC		658/−183	
	IOLR-P374-D-**XI**	CC**TCTAGA**GCAATTGTCGCGATAGAAGA			
*otsB* (750)	OSTB-P4868-A-**XI**	GG**TCTAGA**TGCGCGATCTTCATGAACAA	454/+75		503
	OSTB-P4868-B-**HIII**	TTCATCCGTCAGA**AAGCTT**GTCGGAGCAATGTCGAGAAG			
	OSTB-P4868-C-**HIII**	**AAGCTTT**CTGACGGATGAAGGAATGTT		500/−172	
	OSTB-P4868-D-XI	GG**TCTAGA**ATAGCCGGACATCTCATGCC			
*thuR* (1020)	THUR-P6269-A-**XI**	GG**TCTAGA**GGTCCTCAGAAGCATTGTCA	473/+87		865
	THUR-P6269-B-**HIII**	GTTCGTGAATCTC**AAGCTT**CTCGCTGACCTCGGGATAG			
	THUR-P6269-C-**HIII**	**AAGCTT**GAGATTCACGAACTCTGGCC		453/−69	
	THUR-P6269-D-**XI**	GG**TCTAGA**TAGGTGCGATGAACATGACG			
*nesR* (783)	NESR-P5735-A-**XI**	CGC**TCTAGA**GGCTCGATATCACGCCAC	186/+39		749
	NESR-P5735-B	TTAAGCCGCGGCGGTGAACTGGTTCCTGATC			
	NESR-P5735-C	GCCGCGGCTTAAAACACC		160/+6	
	NESR-P5735-D-**XI**	GCG**TCTAGA**ACGACAAGCGGCAGCTTGC			
*sahR* (1023)	SAHR-P2239-A-**AI**	**GGGCCC**GACAACATCTCGAAGG	1062/+36		973
	SAHR-P2239-B-**PI**	**CTGCAG**AGCATCCAAACCAAGCG			
	SAHR-P2239-C-**PI**	**CTGCAG**GGGGAGTTTGAGAATGA		1132/−14	
	SAHR-P2239-D-**XI**	**TCTAGA**CCGAATTAGGGACTATAATTCCG			

* Bold font indicate restriction sites (XI: XbaI, HIII: HindIII, AI: ApaI, PI: PstI). Red font shows complementary sequences used for the fusion of 5′ and 3′ regions.

## Data Availability

The genome and predicted gene sequences are accessible and can be downloaded from the NMPDR, SEED-based prokaryotic genome annotation service (https://rast.nmpdr.org/rast.cgi) using “guest” as login and password or upon request from the authors.

## Supplementary Materials

The following are available online at https://www.mdpi.com/article/10.3390/microorganisms10020298/s1, Figure S1: Genetic map of the GSR regulatory genes *rsiA1*, *rsiB1* and *rpoE2.*
