# Supplementary material for: Analysis of Ensifer aridi Mutants Affecting Regulation of Methionine, Trehalose, and Inositol Metabolisms Suggests a Role in Stress Adaptation and Symbiosis Development
Source: Microorganisms. 2022 Jan 26;10(2):298. doi: 10.3390/microorganisms10020298 (PMC8877191; doi:10.3390/microorganisms10020298)

Figure S1. Genetic map of the GSR regulatory genes *rsiA1*, *rsiB1* and *rpoE2*. *Ensifer aridi* predicted encoding genes (PEGs) are shown below gene names. The presence of putative IolR and RpoE2 binding sites in the *rsiB1-rsiA1* intergenic sequence are indicated bellow the genetic map as red and brown boxes respectively.

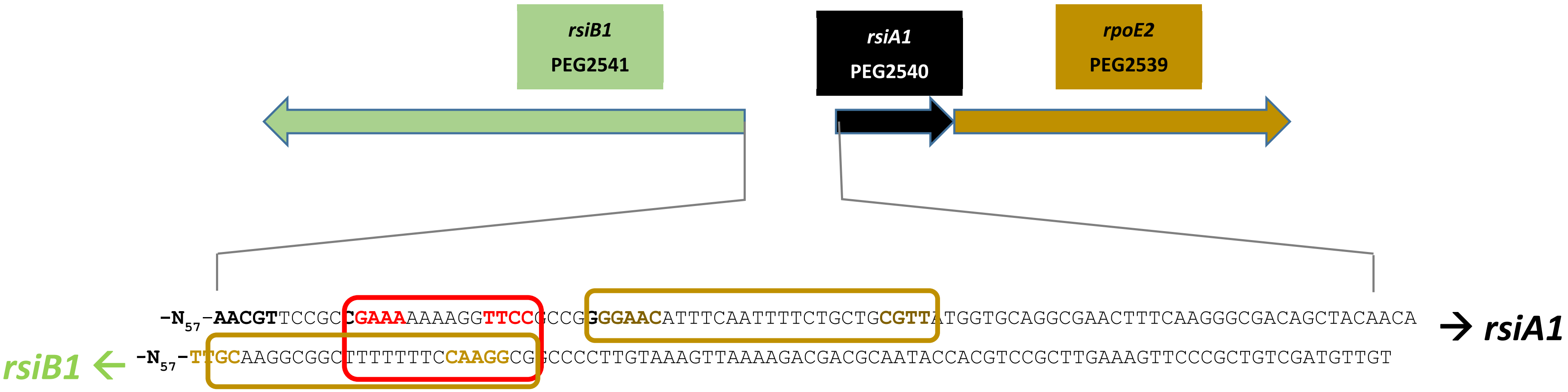

Supplement: Supplementary file 1 [file microorganisms-10-00298-s001.zip › microorganisms-1541148-supplementary.pdf]
